# Disruptive and avoidable: GDPR challenges to secondary research uses of data

**DOI:** 10.1038/s41431-020-0596-x

**Published:** 2020-03-02

**Authors:** David Peloquin, Michael DiMaio, Barbara Bierer, Mark Barnes

**Affiliations:** 1 Ropes & Gray LLP, Boston, MA USA; 2 Harvard Medical School, Boston, MA USA; 3 Ropes & Gray LLP and Yale Law School, Boston, MA USA

**Keywords:** Medical research, Ethics, Health policy, Drug regulation

## Abstract

The advent of the European Union’s General Data Protection Regulation (GDPR) has posed several significant difficulties for the secondary research uses of data and associated biospecimens and has led to widespread unease within the international biobanking and databanking community. This disruption of research using personal data and associated biospecimens has gone largely unremarked in the professional literature, including in a recent account of GDPR’s relationship to biobanking practices published in this journal, which instead advocated even more stringent, and in our view, unnecessary restrictions on research uses of banked data and materials. In this article, we describe challenges that GDPR has posed for biobanks and databanks and for researchers who use those banked resources for secondary research. We discuss the limitations inherent in the few pathways that GDPR makes available for secondary research, given that such pathways rely upon complex and varied laws of individual European Union member states. We advocate mitigation of these difficulties through regulatory guidance in order to allow important scientific research to continue.

## Introduction

The advent of the European Union’s (EU) General Data Protection Regulation (GDPR) has posed major challenges to the secondary research uses of data and associated biospecimens and has led to widespread unease within the international biobanking and databanking community [1]. Oddly, the degree of disruption in research using personal data and associated biospecimens has gone largely unremarked in the professional literature. In our view, GDPR, with its problematic and, in many cases, ill-considered application to secondary research uses of personal data, has unnecessarily complicated the operation of research biobanks and associated data, without appreciably improving privacy protections afforded the human sources of those biospecimens and data. For this reason, we seek to dispel misconceptions regarding the challenges faced under GDPR by biobanks and databanks that conduct secondary research or share biospecimens and data with third parties to conduct research. We describe below several challenges that GDPR has posed for the research community, particularly for the biobanking and databanking community and researchers who seek to deploy those resources for biomedical studies. Finding solutions to these challenges is imperative if the EU is to pursue successfully the ambitious research goals set forth in Horizon Europe and other research initiatives.

## Difficulties under GDPR for secondary research

GDPR imposes several obligations upon the research enterprise, specifically in connection with secondary research uses of personal data. We use the term “secondary research” to refer to research conducted using data or biospecimens collected either: (i) for research studies other than the proposed research or (ii) for nonresearch purposes, such as clinical care. Secondary research is important to biobanks and databanks, which hold biospecimens and accompanying phenotypic and demographic data for distribution to other researchers for secondary research purposes.

GDPR creates confusion for biobank research because of its treatment of “pseudonymized” versus “anonymized” data; its failure to provide a clear legal basis for the processing of personal data for secondary research purposes; and the restrictions placed on the cross-border transfer of personal data from the EU to jurisdictions located outside the EU.^1^ Each of these features of GDPR has profoundly and adversely affected research using biobank and databank resources.

### Pseudonymous and anonymous data

The first challenge created by GDPR stems from its treatment of “anonymous” versus “pseudonymous” data. GDPR’s recitals discuss the concept of anonymized data differently from how the term “anonymous” has historically been understood by the research community. Specifically, data and biospecimens used in secondary research are very often key-coded (that is, assigned unique identifiers in place of readily identifiable information, such as names or national identity numbers). In this way, the research team conducting the secondary research is unable to identify the individual data subjects whose data and specimens are used, because the research team lacks the key needed to reidentify the data and is bound by policy, or by formal agreement, not to seek access to the key from the entity that holds it. The clinician who originally collected the data and biospecimens, or in some cases the biobank that collects the data and biospecimens and replaces directly identifiable information with a code, typically retains a key to the data subject’s identity, which permits resort to primary data in the event of any data integrity questions, and may also, for example, allow the research team to provide to the clinician any important, medically actionable findings discovered during the research that the clinician may wish to return to the data subject.

Under many privacy and research regulatory regimes, including that of the United States, data that have been key-coded in the fashion described above are understood to be “deidentified” or “anonymized,” as applicable, and thus, outside the applicable scope of regulation. For example, under the US Health Insurance Portability and Accountability Act of 1996 (HIPAA), a covered entity or its business associate may deidentify protected health information by either (i) engaging an expert to determine that “the risk is very small that the information could be used, alone or in combination with other reasonably available information, *by an anticipated recipient* to identify an individual who is a subject of the information”; or (ii) removing a set of eighteen identifiers from the data, such as name, all elements of dates directly relating to an individual, and contact information [2]. Notably, HIPAA allows a covered entity to assign a code or other means of record identification to allow deidentified information to be reidentified by the covered entity, provided that: (i) the code or other means of identification is not derived from or related to information about the individual and is not otherwise capable of being translated so as to identify the individual; and (ii) the covered entity does not use or disclose the code or other means of identification for any other purpose and does not disclose the mechanism for reidentification [3]. Similarly, under the national regulations that govern research involving human subjects in the United States, known as the Common Rule, existing data sets are considered to be individually identifiable, and thus, subject to the rule’s protections, only if the identity of the data subject is or may readily be ascertained by the investigator or associated with the information [4]. The information is not regarded, as a legal matter, as readily ascertainable if, for example, the investigators and the holder of the key to coded information agree to prohibit release of the key under any circumstances until the individuals are deceased. Similarly, the United Kingdom’s Information Commissioner’s Office, in its *Code of Practice* on anonymization issued under the GDPR’s predecessor data protection regime, the Data Protection Directive (the “Directive”), took the position that pseudonymous data could be considered anonymized when used for research purposes subject to adequate safeguards [5].

GDPR, on the other hand, states in Recital 26 that “[t]he principles of data protection should apply to any information concerning an identified or identifiable natural person” and further that “[p]ersonal data which have undergone pseudonymisation, which could be attributed to a natural person by the use of additional information should be considered to be information on an identifiable natural person.” Recital 26 continues that “[t]o determine whether a natural person is identifiable, account should be taken of all the means reasonably likely to be used… either by the controller or by another person to identify the natural person directly or indirectly.” This language strongly implies that, contrary to the contextual approach described above under which specimens and data held by researchers are considered not to be “personal data” if the researcher lacks the key needed to reidentify the specimens and data, GDPR takes the position that all pseudonymized data are considered personal data, regardless of whether they are, *or ever will be*, in the hands of a person who holds the key needed for reidentification. There has been limited guidance from the European Data Protection Board (EDPB) further clarifying this recital. The Article 29 Working Party (the predecessor to the present EDPB) issued guidance on anonymization and pseudonymization, but the guidance predated GDPR and has not yet been adopted by the EDPB [6].

Notably, at least one regulatory authority, the United Kingdom’s Health Research Authority (UK-HRA), has suggested that GDPR should be interpreted consistent with prior practice in the United Kingdom to consider pseudonymized data not to be personal data when held by someone who is prohibited from receiving the key needed for reidentification [7]. At least at present, however, the UK-HRA position appears to be an outlier among EU member states. Moreover, given the United Kingdom’s recent departure from the EU, it appears likely that the view of United Kingdom bodies will be given less weight in future interpretations of GDPR.

Interestingly, the EU-US Privacy Shield program, which permits the transfer of personal data from the EU to businesses in the US that have self certified to the Privacy Shield program, takes a similar approach to that of the UK-HRA on this issue, stating as follows:

Invariably, research data are uniquely key-coded at their origin by the principal investigator so as not to reveal the identity of individual data subjects. Pharmaceutical companies sponsoring such research do not receive the key. The unique key code is held only by the researcher, so that he or she can identify the research subject under special circumstances (e.g., if follow-up medical attention is required). A transfer from the EU to the United States of data coded in this way would not constitute a transfer of personal data that would be subject to the Privacy Shield Principles [8].

This statement in the Privacy Shield Principles, although helpful to secondary use research, is not able to be relied upon by a research entity in the Privacy Shield framework, given that the Privacy Shield language predated GDPR, and given the disparate approaches under Recital 26 of GDPR and under EU member state interpretations thereof. Moreover, the Privacy Shield is available only to US-based for-profit entities, and not to not-for-profit universities and medical research entities.

The Court of Justice of the European Union (CJEU) addressed the scope of the definition of personal data in *Patrick Breyer v. Bundesrepublik Deutschland*. While this case was decided under the Directive and not under GDPR, given the similarity in the definition of personal data found in the Directive to that found in GDPR, the case remains instructive in interpreting the term personal data. In *Breyer*, the court reviewed whether a dynamic IP address held by a controller should be considered personal data, where such data could not be used to identify a user standing alone, but could identify a user when combined with additional data held by the user’s internet service provider. The CJEU reasoned that the data would not be identifiable if identification were “prohibited by law or practically impossible” due to “a disproportionate effort in terms of time, cost and man-power, so that the risk of identification appears in reality to be insignificant.” Nevertheless, in that case, the court found the data to be identifiable because the controller had the “legal means” to identify the user, even though the legal means were available only in the narrow event of a cyber attack warranting notification to individual users [9]. Although the CJEU thus did not find the data to be anonymous in *Breyer*, the CJEU suggested that legal constraints on access to the key to reidentify key-coded data could render data anonymized rather than pseudonymized, under certain circumstances.

This contextual approach to anonymization taken by the UK-HRA and reflected in the Privacy Shield framework and the *Breyer* decision would appear to be an eminently reasonable interpretation of GDPR provisions from the standpoint of the research community. Elsewhere, GDPR pursues a context-driven and scalable approach, as reflected in GDPR requirements related to data security (requiring implementation of measures “appropriate to the risk”), data protection impact assessments (requiring assessments of risks to rights and freedoms of natural persons), and appointments of data protection officers (requiring determination of whether processing is “core” to the entity and whether processing is performed on a “large scale”). (GDPR Art. 32(1); Art. 35 (1); Art. 37(1)). Moreover, deeming pseudonymized data as personal data without review of the context results in a limitless definition of personal data that requires compliance with GDPR regardless of how low the possible risk would be to the individuals involved. In interpreting Recital 26, emphasis should be placed on the “means *reasonably* likely to be used,” and the circumstances of the ability to reidentify by a given researcher should be taken into account.

However, under the prevailing regulatory authorities’ GDPR interpretation of pseudonymized data as identifiable personal data under Recital 26, GDPR has expanded the reach of privacy law through its direct regulation of data that were previously regarded for research purposes as not constituting protected personal data. Key-coded data sets are therefore no longer anonymized and must be treated as though they are fully identifiable data, subject to all requirements for fully identifiable data. The practical effect of this treatment is that biobanks that lack the ability to link key-coded data to the data subject’s identity and that therefore previously took the position that the data they hold are anonymous and thus outside of privacy law regimes are now subject, in regard to their data use practices, to direct regulation under GDPR. Similarly, researchers who access biobanked materials and their associated data also cannot take the position that key-coded data are not personal data. This resulting sea change in the treatment of pseudonymized data has not stemmed from any apparent misuse of key-coded data, or breach of privacy, by biobanks or researchers, yet it has effectively imposed a new compliance obstacle to routine banking and secondary uses of key-coded personal data. Now that effectively all data held and distributed by biobanks and databanks, even though key-coded, are treated as fully identifiable data under GDPR, the research use of those data and associated biospecimens must have a legal basis in the same way as is required for use of data and associated biospecimens that bear names of their human sources. Researchers conducting secondary research on health or genetic data (who, in GDPR parlance, are “controllers” of those data) therefore must demonstrate a lawful basis to process personal data under Article 6 of GDPR and an exception to allow processing of “special categories of personal data,” such as health or genetic data, under Article 9 of GDPR. Yet explicit consent (GDPR Art. 6(1)(a); Art. 9(2)(a)) most often is not a feasible basis for processing personal data for secondary research because the controller researcher does not know, and cannot obtain, the identity of data subjects in data sets received from biobanks that hold only pseudonymized data.

### Challenges regarding consent

Perhaps, one might estimate, challenges created by the expanded interpretation of the definition of “personal data” could be solved by seeking from data subjects a form of broad consent for future research uses of their personal data. Indeed, GDPR’s legislative history endorses such an approach, but here too, EU and member state regulatory agency interpretations have foreclosed any such “broad consent” solution to allow research uses of personal data, even key-coded, otherwise fully anonymized data, in biobanks. Specifically, GDPR Recital 33 suggests the use of a broad consent from data subjects to facilitate secondary uses of personal data for research:

It is often not possible to fully identify the purpose of personal data processing for scientific research purposes at the time of data collection. Therefore, data subjects should be allowed to give their consent to certain areas of scientific research when in keeping with recognized ethical standards for scientific research. Data subjects should have the opportunity to give their consent only to certain areas of research or research projects to the extent allowed by the intended purpose. (GDPR, Recital 33).

Indeed, this would appear to offer a viable option to facilitate secondary research uses of identifiable data and associated biospecimens. However, subsequent guidance on consent from the Article 29 Working Party severely limited any possible salutary interpretation of Recital 33, by essentially eviscerating any ability to obtain broad research consent from human sources of personal data and biospecimens. This evisceration is accomplished by the Article 29 Working Party’s indicating, in official guidance, that “special categories” of data, such as health or genetic data, can be used only pursuant to consent that is very specific as to a future use, and not broad in nature.

Recital 33 does not disapply the obligations with regard to the requirement of specific consent. This means that, in principle, scientific research projects can only include personal data on the basis of consent if they have a well-described purpose. For the cases where purposes for data processing within a scientific research project cannot be specified at the outset, Recital 33 allows as an exception that the purpose may be described at a more general level. Considering the strict conditions stated by Article 9 GDPR regarding the processing of special categories of data, WP29 notes that when special categories of data are processed on the basis of explicit consent, applying the flexible approach of Recital 33 will be subject to a stricter interpretation and requires a high degree of scrutiny. When regarded as a whole, the GDPR cannot be interpreted to allow for a controller to navigate around the key principle of specifying purposes for which consent of the data subject is asked [10].

This EU guidance interpreting and limiting the use of broad consent for research has significantly narrowed the extent to which consent could ever be a realistic basis for processing in the context of banked personal data and associated biospecimens.

Yet even very specific consent for a future secondary research use of personal data has come under pressure from the EU authorities. The EDPB has continued to express skepticism regarding the use of consent as the basis for processing personal data for research purposes, noting in a 2019 guidance document on the intersection of GDPR and the EU Clinical Trials Regulation (CTR) that consent should not be the basis relied upon for processing of personal data in the context of a clinical trial, given that the “power imbalance” between the investigator and data subject prevents consent from being “freely given” [11]. This is a very curious—even anomalous—result, given that the EU CTR typically requires the consent of the subject for enrollment in the clinical trial despite the same power dynamics inherent to the clinical trials context. Thus, it is not clear how the consent to enter the trial and take an investigational product whose safety and efficacy are unproven can be “freely given” but consent to process one’s data in connection with such a trial cannot be “freely given.”

### Exceptions for research under GDPR and member state law

One may then suspect that as a comprehensive privacy regime, GDPR would include some category of special exceptions to allow processing of personal data, including health data, for scientific research, thus avoiding the consent and broad consent issues altogether. Indeed, GDPR allows for such exceptions both within GDPR itself as well as the ability for individual EU member states to create “derogations” (exceptions under member state law) for research. However, as explained herein, none of these exceptions is adequate, logistically or for compliance purposes, in the case of secondary use of personal data, including personal data accessed from biobanks or databanks. Specifically, GDPR allows processing of special categories of personal data when “necessary for reasons of public interest in the area of public health, such as… ensuring high standards of quality and safety of health care and of medicinal products or medical devices, on the basis of Union or Member State law which provides for suitable and specific measures to safeguard the rights and freedoms of the data subject, in particular professional secrecy” (GDPR, Art. 9(2)(i)) as well as when “necessary for archiving purposes in the public interest, scientific or historical research purposes… in accordance with Article 89(1) based on Union or Member State law which shall be proportionate to the aim pursued, respect the essence of the right to data protection and provide for suitable and specific measures to safeguard the fundamental rights and the interests of the data subject.” (GDPR, Art. 9(2)(j)). Further, GDPR allows for processing of personal data when such processing is not “incompatible” with the initial purposes of processing, noting that “further processing for archiving purposes in the public interest, scientific or historical research purposes or statistical purposes shall, in accordance with Article 89(1), not be considered to be incompatible with the initial purposes.” If conducting research under Article 9(2)(j) or as compatible with the initial purposes, the research must be conducted consistent with the technical and organizational measures under Article 89(1), including data minimization, and “may include pseudonymisation provided that the purposes can be fulfilled in that manner.”

Yet these several exceptions have proven, in practice, elusive. When processing based on “public interest in the area of public health” or “archiving purposes in the public interest, scientific or historical research purposes,” controllers must do so based on EU or member state law. Notably, however, implementing laws in several EU member states do not clearly permit processing for secondary research under these provisions of GDPR. We understand that the European Commission intends to survey EU member state laws regarding this topic in the near future. Similarly, while the ability to process personal data for “compatible” purposes at first appears promising to the biobanking and databanking community, there has been no effective guidance issued on this provision to date regarding secondary research use of personal data, and the EDPB has stated that it needs to examine this provision and issue further guidance in due course [12].

Thus, at the same time GDPR has expanded the application of privacy law to biobanks by including “pseudonymized” data in the definition of personal data, GDPR has left the biobank community and researchers with no clear basis on which to rely for the processing of personal data: on the one hand, the guidance issued to date has expressed skepticism regarding the use of broad consent for processing of personal data for research purposes, but on the other hand, no clear alternative that can be applied consistently across EU member states has been identified. This has caused confusion in the research community and has led to a reluctance or outright refusal on the part of many research institutions to share data and specimens for secondary research.

### Cross-border transfers of personal data

In addition to the challenges discussed above, which are applicable to both intra-European and ex-European research, GDPR imposes additional challenges for research collaborations between researchers located in the EU and those located outside the EU. Specifically, GDPR requires that any controller or processor transferring personal data from within the EU to a jurisdiction outside the EU that the European Commission has not deemed adequately to protect personal data must demonstrate a lawful basis for making the transfer. This is separate from the requirement discussed above to establish a basis for processing personal data. At present, the European Commission has recognized only eight non-EU countries as providing adequate data protection, and the USA is not among them.^2^

The lawful bases under GDPR for cross-border transfer of personal data are limited with respect to secondary research. Typical bases to transfer personal data in the research context include standard contractual clauses approved by the European Commission or consent of the data subjects. Consent often is not a viable option for cross-border transfer of personal data from biobanks for secondary research, however, because data subjects often are not in direct contact with the biobank or databank holding their data, or researchers who are poised to use their data, and thus the biobank and researchers would not be able to contact the data subject to obtain that person’s consent to transfer personal data to an “insecure” jurisdiction. The default approach in such situations is thus often standard contractual clauses, which are form agreements that, by law, cannot be altered.

However, the standard contractual clauses are not viable when the recipient entity is an arm of the US government, such as the US National Institutes of Health (NIH) or public universities or academic medical centers, because the US government cannot agree to certain terms in the standard contractual clauses including dispute resolution in European courts, and US state entities (including state universities and public hospitals) often similarly cannot agree to certain terms due to restrictions in their state or local laws. Given the large amount of international biobank research, including research occurring in the EU, that is funded by the US NIH or that involves US public universities or academic medical centers, the inability to rely upon the standard contractual clauses has proved a major obstacle to much secondary research.

Finally, ambiguity regarding the aggressive—and indeed nearly unparalleled—broad territorial scope of GDPR presents difficulties for biobanks facilitating secondary research and for researchers seeking to use the biobank data. One of the most novel aspects of GDPR is its extraterritorial scope, which is more expansive than that found in most previous privacy laws. GDPR applies not only to entities that are “established” in the EU, but also to entities that process personal data in relation to (i) offering goods or services to data subjects located in the EU, or (ii) monitoring behavior of persons located in the EU. It is, at present, not fully clear if a US or other ex-EU biobank that processes specimens and accompanying phenotypic data from the EU would be considered to be offering goods or services to the persons whose data are included in the biobank or monitoring the behavior of such persons. Arguably, if no research results are returned to the research subjects, no good or service is being offered to them, and GDPR consequently would not apply. If, however, personal data are provided to the ex-EU biobank or databank on a periodic or “real-time” basis, it could be argued that the behavior of the data subjects to whom the data pertain is being monitored by the ex-EU biobank or researchers. The lack of clarity on this point has further complicated transnational biobanking, databanking, and secondary research activities. Although the EDPB has issued territorial scope guidelines, which discuss when the monitoring behavior prong would be triggered, the guidelines speak generally to the idea that GDPR could apply through “monitoring or regular reporting on an individual’s health status,” without specific discussion of the situations faced by ex-EU biobanks and databanks or researchers in the biomedical context [13].

Further guidance would be helpful to narrow the complexity surrounding processing of personal data for secondary research. Not all EU member states have implemented provisions related to research, and among the member states that have done so, they have varied in terms of additional obligations for the research community, preferred bases for processing, and available derogations upon rights requests. Uniform guidance issued by the EDPB on the challenges discussed above would be beneficial in providing a more consistent and clearer set of obligations, and there appears to be room for legitimate interpretation of GDPR standards that would alleviate some or all of the difficulties described above.

## GDPR expands existing obligations for the research community

Recently, in this journal, an account of GDPR’s effects on biobanking practices failed to relate the difficulties faced under GDPR for biobanks, databanks and secondary researchers, and, moreover, advocated even more stringent restrictions on research uses of banked data and materials. Specifically, in the article entitled *The GDPR and the Research Exemption: Considerations on the Necessary Safeguards for Research Biobanks*, Staunton, Slokenberga, and Mascalzoni compare the obligations for research biobanks under GDPR to ethical obligations for research that exist under a variety of European and international legal instruments that regulate research on health data [14]. Based on their review, the authors argued that GDPR insufficiently protects individual rights of data subjects, in part because GDPR allows member states to enact derogations to permit processing of personal data for research purposes without subject consent and allows member states to limit the ability of data subjects to exercise certain rights with respect to their data when the data are processed for research purposes. The authors expressed concern regarding erosion of protection of research subjects if data are processed for secondary research purposes on the basis of GDPR’s scientific research or public health exceptions. Yet this line of argument seems to promote, and rely for research participant protection on, privacy law, as opposed to the parallel regime of human subjects research regulations. Indeed, even if a researcher relies upon the GDPR’s scientific research or public health exceptions, there remain separate rigorous ethical and legal constraints related to research that protect the rights and interests of research subjects. Staunton et al. express further concern regarding the potential for individual EU member states to grant derogations to the obligations of individual rights in the research context. Yet there are valid and well-recognized rationales for granting derogations of data subject rights for essential research that would not otherwise be reasonably possible to conduct.

### Ongoing ethical obligations in research

The recitals to GDPR make clear that GDPR was not intended to replace the existing obligations to obtain informed consent under the EU CTR and EU member state laws. Specifically, Article 28(2) of CTR requires that sponsors obtain subject consent for secondary research with data collected in a primary clinical trial. The recitals to GDPR discuss the continuing obligations that other laws place on researchers, noting as follows in Recital 157, with emphasis added by the authors to note the explicit reference to other conditions and safeguards under member state law:

By coupling information from registries, researchers can obtain new knowledge of great value with regard to widespread medical conditions such as cardiovascular disease, cancer and depression. On the basis of registries, research results can be enhanced, as they draw on a larger population. Within social science, research on the basis of registries enables researchers to obtain essential knowledge about the long-term correlation of a number of social conditions such as unemployment and education with other life conditions. Research results obtained through registries provide solid, high-quality knowledge that can provide the basis for the formulation and implementation of knowledge-based policy, improve the quality of life for a number of people and improve the efficiency of social services. *In order to facilitate scientific research, personal data can be processed for scientific research purposes, subject to appropriate conditions and safeguards set out in Union or Member State law*.

Given GDPR’s requirement that research must be conducted in accordance with additional safeguards in EU or member state law, concern that processing personal data for scientific research would take place in contravention of these existing laws covering human subjects research is unfounded. Moreover, as noted above, GDPR requires that processing for research purposes be conducted consistent with the technical and organizational measures found in GDPR Article 89(1), including data minimization and pseudonymization when possible, which offer significant protection for personal data privacy.

### Rights limitations in the research context

Another concern articulated by Staunton et al. is that GDPR’s allowing EU member states to grant derogations to data subject rights when data are processed for research undermines protections for data subjects. However, there are multiple reasons why a privacy regime should not grant absolute rights to research subjects with respect to the processing of their personal data for research purposes.

Typically, the data controller (the biobank, databank or researcher) in such scenarios will only have access to pseudonymized personal data for performance of the research. Further, the controller typically will lack the key to reidentify the personal data involved and as noted above, may be bound by policy, or by formal agreement, not to seek access to the key from the entity that holds it or to seek access to the key only in extremely limited circumstances, such as to investigate an allegation of research misconduct. Thus, logistically, it may not be possible to afford data subjects any rights of access to their data or to the erasure of their data: due to the key-coded way in which their personal data are housed, their unique data could not even be identified by the controller.

Moreover, even in situations when it may be possible to grant data subject rights, ethical and legal obligations to ensure proper reporting of research outcomes require that the data set involved in a study be maintained for future reference purposes. Granting the right of erasure, as well as, in some instances, the right to object to further processing, could nullify the research involved. GDPR recognizes that the existing regulatory frameworks surrounding research have functioned appropriately regarding individual rights related to research, and therefore GDPR defers for these purposes to those more tailored frameworks. The derogations of data subject rights in the research context would therefore seem a decidedly positive aspect of GDPR. Expanding a right of erasure to be more widely available to research subjects risks corroding and impeding future research and does not add any effective actual protections to research subjects, whose privacy interests are already protected under existing biobanking practices, as discussed above.

## Conclusion

GDPR presents several significant difficulties for biobanking and databanking, including failing to provide a clear basis for processing personal data for secondary research purposes. The few regulatory pathways that GDPR provides lead to complex variations among EU member states, and these variations add significant transaction costs and barriers to secondary research uses of data and biospecimens. To the extent that GDPR does not speak to all ethical requirements related to research, the regulation’s drafters did not set out to do so, and instead, properly recognized that the existing ethical and legal framework for research with humans should continue to guide the research community. Because the GDPR was intended as a law of general applicability that would offer protection to personal data when processed in all sectors of the EU economy, the unique challenges it has created for the research enterprise were likely unanticipated and unintended. This makes it more likely that solutions now could be devised to ease the use of personal data for important biomedical research while maintaining adequate protections on data subject privacy. Although EDPB guidance is not binding legal authority and would be overridden by interpretations in EU member states to the contrary, EDPB guidance is given a great deal of deference in practice. This is, in part, because EDPB is comprised of representatives of each member state and provides the best reading on supervisory authorities’ understanding of GDPR obligations. Further guidance from the EDPB would be beneficial in areas where GDPR has created hurdles for the research community, specifically regarding: (i) the concept of anonymization, specifically whether key-coded data can be considered anonymized data under certain circumstances; (ii) the basis for processing personal data for secondary research purposes; and (iii) the basis for cross-border transfer of personal data for research purposes. Indeed, the European Data Protection Supervisor has recognized the need for further guidance in a January 2020 preliminary opinion regarding the relationship between data protection and scientific research [15]. We hope that this preliminary opinion leads to further guidance from the EDPB.

## Footnotes

1 The GDPR applies to the member states of the European Economic Area, which include the EU as well as Iceland, Liechtenstein and Norway. We refer to EU through the article for simplicity.

2 The European Commission has recognized Andorra, Argentina, Canada (commercial organizations), Israel, Japan, New Zealand, Switzerland, and Uruguay as adequately protecting data. The European Commission has also recognized the British Crown Dependencies of Guernsey, the Isle of Man, and Jersey as offering adequate data protection, as well as the Faroe Islands, which is an autonomous territory of the Kingdom of Denmark.

## References

[1] Regulation (EU) 2016/679 of the European Parliament and of the Council of 27 April 2016 on the protection of natural persons with regard to the processing of personal data and on the free movement of such data, and repealing Directive 95/46/EC (General Data Protection Regulation).

[2] 42 Code of Federal Regulations § 164.514(b).

[3] 42 Code of Federal Regulations § 164.502(d); US Department of Health and Human Services, Office of Civil Rights, Guidance Regarding Methods for De-identification of Protected Health Information in Accordance with the Health Insurance Portability and Accountability Act (HIPAA) Privacy Rule, https://www.hhs.gov/hipaa/for-professionals/privacy/special-topics/de-identification/index.html.

[4] US Department of Health and Human Services, Office for Human Research Protections, Coded Private Information or Specimens Use in Research, Guidance (2008), https://www.hhs.gov/ohrp/regulations-and-policy/guidance/research-involving-coded-private-information/index.html.

[5] United Kingdom Information Commissioner’s Office, Anonymisation: Managing Data Protection Risk Code of Practice (Nov 2012), Annex 1, (noting that information that has been pseudonymized through use of a key is not personal data in the hands of a researcher who lacks access to the key), https://ico.org.uk/media/1061/anonymisation-code.pdf.

[6] Article 29 Working Party, Opinion 05/2014 on Anonymisation Techniques (WP216) (April 10, 2014), https://iapp.org/media/pdf/resource_center/wp216_Anonymisation-Techniques_04-2014.pdf.

[7] National Health Service Health Research Authority, Controllers and personal data in health and care research, (19 April 2018), https://www.hra.nhs.uk/planning-and-improving-research/policies-standards-legislation/data-protection-and-information-governance/gdpr-guidance/what-law-says/data-controllers-and-personal-data-health-and-care-research-context/.

[8] Privacy Shield Framework, § III.14.g, Pharmaceutical and Medical Products: Key-coded Data (2016), https://www.privacyshield.gov/article?id=14-Pharmaceutical-and-Medical-Products.

[9] Court of Justice of the European Union, Patrick Breyer v. Bundesrepublik Deutschland (C-582/14), § 44-46; 49 (19 Oct 2016).

[10] Article 29 Working Party, Guidelines on Consent under Regulation 2016/679 (WP259rev.01), pp 28 (rev. 10 April 2018), https://ec.europa.eu/newsroom/article29/item-detail.cfm?item_id=623051.

[11] European Data Protection Board, Opinion 3/2019 Concerning the Questions and Answers on the Interplay Between the Clinical Trials Regulation (CTR) and the General Data Protection Regulation (GDPR) (23 Jan 2019), https://edpb.europa.eu/our-work-tools/our-documents/avis-art-70/opinion-32019-concerning-questions-and-answers-interplay_en.

[12] *See* n 11, p 8. Specifically, the EDPB states: “These conditions [secondary research], due to their horizontal and complex nature, will require specific attention and guidance from the EDPB in the future. For the time being, the presumption of compatibility, subject to the conditions set forth in Article 89, should not be excluded, in all circumstances, for the secondary use of clinical trial data outside the clinical trial protocol for other scientific purposes.”

[13] European Data Protection Board, Guidelines 3/2018 on the Territorial Scope of the GDPR (Article 3) – Version 2.0 (12 Nov 2019), https://edpb.europa.eu/sites/edpb/files/files/file1/edpb_guidelines_3_2018_territorial_scope_after_public_consultation_en.pdf.

[14] Staunton C, Slokenberga S, Mascalzoni D. The GDPR and the Research Exemption: Considerations on the Necessary Safeguards for Research Biobanks. Eur J Hum Genetics. 2019; e-pub ahead of print 17 April 2019; 27:1159–1167, https://www.nature.com/articles/s41431-019-0386-5.pdf. 10.1038/s41431-019-0386-5 PMC6777499 30996335

[15] European Data Protection Supervisor, A Preliminary Opinion on Data Protection and Scientific Research (6 Jan 2020), https://edps.europa.eu/sites/edp/files/publication/20-01-06_opinion_research_en.pdf.

